# Inhibition of nitric oxide synthase unmasks vigorous vasoconstriction in established pulmonary arterial hypertension

**DOI:** 10.14814/phy2.13537

**Published:** 2017-12-06

**Authors:** Mariko Tanaka, Kohtaro Abe, Masahiko Oka, Keita Saku, Keimei Yoshida, Tomohito Ishikawa, Ivan F. McMurtry, Kenji Sunagawa, Sumio Hoka, Hiroyuki Tsutsui

**Affiliations:** ^1^ Department of Cardiovascular Medicine Kyushu University Graduate School of Medical Sciences Fukuoka Japan; ^2^ Department of Anesthesiology and Critical Care Medicine Kyushu University Graduate School of Medical Sciences Fukuoka Japan; ^3^ Departments of Pharmacology and Internal Medicine, and Center for Lung Biology University of South Alabama Mobile Mobile Alabama; ^4^ Department of Therapeutic Regulation of Cardiovascular Homeostasis Center for Disruptive Cardiovascular Medicine Kyushu University Fukuoka Japan

**Keywords:** Endothelial function, L‐NAME, nitric oxide, pulmonary arterial hypertension

## Abstract

It is widely accepted that impaired bioavailability of endothelial nitric oxide (NO) plays a critical role in the pathophysiology of pulmonary arterial hypertension (PAH). However, there are published data that show that relatively many PAH patients respond favorably to acetylcholine‐induced pulmonary vasodilation during their follow‐up period, when diverse stages of the disorder are included. We hypothesized that NO bioavailability varies depending on the progression of PAH. Adult rats were exposed to the VEGF receptor blocker Sugen5416 and 3 weeks of hypoxia followed by return to normoxia for various additional weeks. All rats developed increased right ventricular systolic pressure (RVSP) and occlusive lesion formation at 1, 3, 5, and 8 weeks after the Sugen5416 injection. Acute NO synthase blockade did not change the elevated RVSP at the 1‐week time point, while it further increased RVSP markedly at the 3‐, 5‐, and 8‐week time points, leading to death in all rats tested at 8 weeks. Acetylcholine caused significant reduction in RVSP at the 8‐week but not the 1‐week time point, whereas sodium nitroprusside decreased the pressure similarly at both time points. Increased NO‐mediated cGMP production was found in lungs from the 8‐week but not the 1‐week time point. In conclusion, despite its initial impairment, NO bioavailability is restored and endogenous NO plays a critical protective role by counteracting severe pulmonary vasoconstriction in established stages of PAH in the Sugen5416/hypoxia/normoxia‐exposed rats. Our results provide solid pharmacological evidence for a major contribution of a NO‐suppressed vasoconstrictor component in the pathophysiology of established PAH.

## Introduction

Pulmonary arterial hypertension (PAH, Group I pulmonary hypertension) is a diverse group of diseases characterized by narrowing of small pulmonary arteries and arterioles, which results in increased pulmonary vascular resistance and pressure, frequently leading to right heart failure (Tuder et al. 2009). Despite recent advances in treatments, severe PAH remains debilitating and fatal (Humbert et al. 2006). Thus, a better understanding of the complex pathophysiology of PAH is needed for the development of more effective therapies.

Nitric oxide (NO) is well documented to possess potent cardiovascular protective effects by moderating vascular smooth muscle contraction and inhibiting platelet aggregation and cell proliferation (Moncada et al. 1991). Endothelial dysfunction, particularly impaired bioavailability of endothelium‐derived NO (EDNO), has been widely regarded as a central player in the pathogenesis/pathophysiology of PAH (Budhiraja et al. 2004). There are indeed clinical reports that support this logical concept, for example, reduced expression of endothelial NO synthase (eNOS) in lungs and impaired response to endothelium‐dependent vasodilators in isolated pulmonary arteries from PAH patients (Giaid and Saleh 1995; Do e et al. 2009), and decreased levels of exhaled NO in PAH patients (Girgis et al. 2005), although opposing reports against this concept exist (Mason et al. 1998). Also, although numerous experiments have been performed to determine the role of EDNO in the pathogenesis/pathophysiology of PAH using conventional animal models of pulmonary hypertension (PH), that is, chronic hypoxia‐ and monocrotaline‐induced PH, results are mixed and no solid consensus has been made (Adnot et al. 1991; Oka et al. 1993; Xue and Johns 1996; Tyler et al. 1999; Mam et al. 2010).

It is apparent that there are major limitations in determining functional roles of EDNO in the pathophysiology of PAH. For instance, for preclinical animal experiments, there have been no appropriate models that adequately simulate PAH phenotype, that is, severe, progressive PH with formation of plexogenic arteriopathy (Abe et al. 2010), and thus it has been difficult to translate the results obtained using the conventional models to human PAH. For clinical studies, the human data cannot be simply interpreted with regard to their pathophysiological significance, because most data are obtained with tissue/organ samples from patients with PAH at autopsy or lung transplantation when the disorder has advanced to its end‐stage, and the patients likely have received multiple treatments, which complicates the interpretation of the findings. In addition, techniques that assess the functional vascular roles of EDNO clinically are very limited. Finally, one critical aspect that is lacking in the assessment of EDNO is possible temporal changes in its pathophysiological roles. For example, decreased eNOS expression in end‐stage PAH lungs may be real, but data regarding eNOS including its expression and functional roles (e.g., hemodynamic effects of NOS inhibition) at the time of diagnosis when they matter most are extremely limited.

In this regard, one set of clinical data that caught our attention was previously published reports that show frequent favorable responses to the endothelium‐dependent vasodilator, acetylcholine (ACh), that is, significant reductions in pulmonary arterial pressure (PAP) and vascular resistance (PVR), were observed in patients with primary and congenital heart disease associated PH at the time of diagnosis or during their follow‐up period (Marshall et al. 1959). For instance, there are case reports that show marked reductions in PAP and PVR in response to ACh in patients with idiopathic PAH, who were diagnosed with typical symptoms suggesting that the timing of diagnosis were neither very early nor late (Marshall et al. 1959; Satyanarayana Rao et al. 1969). Also, Palevsky et al. (1990) and Shepherd et al. (1959), respectively, found reductions in both PAP and PVR in 12 out of 23 (52%) patients with primary PH and 9 out of 11 (82%) PAH patients with atrial or ventricular septal defects. Because it is reasonably assumed that the patients’ progression status is diverse in these studies, we interpreted these data that there might be a stage or stages during PAH progression when endogenous NO bioavailability is maintained. Based on these clinical data, we hypothesized that NO bioavailability might vary depending on the progression of PAH.

To test this hypothesis, we investigated temporal changes in acute hemodynamic effects of NOS inhibition by *N*_*ω*_‐nitro‐ L‐arginine methyl ester hydrochloride (L‐NAME) and stimulation by ACh in vivo, and eNOS activity (phosphorylated‐eNOS expression) and cGMP content in lungs. The preclinical animal model used in this study was a two‐hit rat model of PAH that possesses major features of human PAH, including development of plexogenic arteriopathy and chronic progressive hemodynamic deterioration (Abe et al. 2010), and has been regarded as a more appropriate preclinical model than the conventional models (Ryan et al. 2011).

## Materials and Methods

### Animals

All experimental procedures were approved by the Institutional Animal Care and Use Committee of Kyushu University, Japan, and all animal procedures were performed by following the principles of the NIH Guide for the Care and Use of Laboratory Animals (NIH Publication, 8th Edition, 2011).

Adult male Sprague‐Dawley rats (150–200 g, Japan SLC, Hamamatsu, Japan) were given a single subcutaneous injection of vascular endothelial growth factor (VEGF) receptor blocker, Sugen5416 (20 mg/kg, Tocris Bioscience, Missouri, United Kingdom) and exposed to hypoxia (10% O_2_) for 3 weeks (wks). They were then returned to normoxia for up to an additional 5 weeks (total 8 weeks after the Sugen5416 injection) (Abe et al. 2010). We examined normal control and four groups (1, 3, 5, and 8 weeks after the Sugen5416 injection) of Sugen5416/hypoxia/normoxia‐exposed (SU/Hx/Nx) rats.

### Drugs

Sugen5416 was suspended in carboxymethylcellulose (0.5% [wt/vol] carboxymethylcellulose sodium, 0.9% [wt/vol] NaCl, 0.4% [vol/vol] polysorbate, 0.9% [vol/vol] benzyl alcohol in deionized water). *N*_*ω*_‐nitro‐ L‐arginine methyl ester hydrochloride (L‐NAME, non‐selective NOS inhibitor), L‐canavanine (selective inducible NOS (iNOS) inhibitor), acetylcholine chloride (ACh, endothelium‐dependent vasodilator), and sodium nitroprusside (SNP, endothelium‐independent vasodilator) were dissolved in saline. All chemicals were purchased from Sigma Aldrich (St. Louis, Mo).

### Catheterized rats

Hemodynamic parameters were evaluated as described previously (Abe et al. 2010; Toba et al. 2014). Briefly, a rat was anesthetized with an intraperitoneal injection of medetomidine hydrochloride (0.15 mg/kg), midazolam (2.0 mg/kg), and butorphanol (2.5 mg/kg) (Tsubokura et al. 2016). An 18‐gauge BD Angiocath catheter (Becton Dickinson) with the tip at a 30‐degree angle was inserted into the right jugular vein and advanced into the right ventricle (RV) for measurement of RV systolic pressure (RVSP) as a marker of systolic pulmonary arterial pressure (PAP) (Schwenke et al. 2011). The catheter was connected to the fluid filled transducer (DX‐360, Nihon Kohden, Japan). A microtip P‐V catheter (FTH‐1912B‐8018, Transonic Inc., Ithaca, NY) was inserted into the right carotid artery and then advanced into the left ventricle (LV). The RVSP, left ventricle systolic pressure (LVSP), heart rate (HR), and cardiac output (CO) were continuously recorded using ML880/9 PowerLab 16/30 (AD Instruments, Dunedin, New Zealand), an ADVantage P‐V control unit (v 5.0) (FY097B, Transonic Inc.) and a dedicated laboratory computer system. Cardiac index (CI) was calculated by dividing CO by body weight. The ratio of RVSP to CI (RVSP/CI) yielded a surrogate marker of total pulmonary vascular resistance index (TPRI) (Pacher et al. 2008; Abe et al. 2010). The ratio of LVSP to CI (LVSP/CI) was also calculated as a surrogate index of systemic vascular resistance (SVRI) (Abe et al. 2010; Toba et al. 2014). After hemodynamic measurements, all rats were euthanized by an overdose of pentobarbital sodium. The heart was removed for assessment of RV hypertrophy (RVH), and the lungs were collected for histological evaluation, immunoblot analysis, and enzyme immunoassay analysis. RVH was expressed as a ratio of the RV to the left ventricle plus septum weight (RV/LV + S) (Abe et al. 2010).

### Experimental protocols in vivo

#### Protocol 1. Role of basal NO release in the normal and hypertensive pulmonary circulation

**Figure 1:**
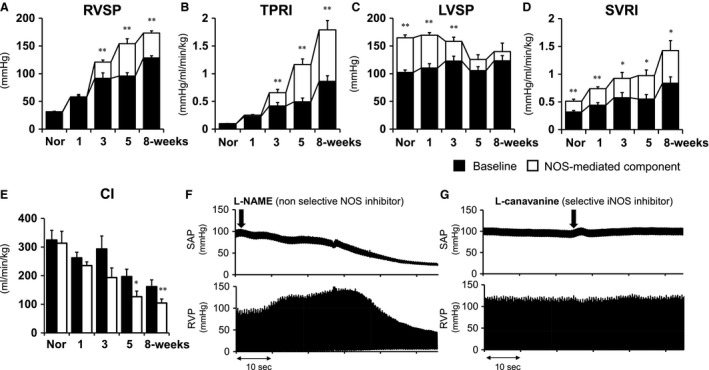
Effects of *N*_*ω*_‐nitro‐ L‐arginine methyl ester hydrochloride (L‐NAME, 30 mg/kg, iv) on hemodynamic parameters in normal (Nor) and Sugen5416/hypoxia/normoxia‐exposed rats at 1, 3, 5, and 8 weeks after the Sugen5416 injection. Black and white columns in each bar indicate values of baseline and changes from baseline after L‐NAME injection (NOS‐mediated component), respectively. Right ventricular systolic pressure (RVSP, (A), total pulmonary vascular resistance index (TPRI, (B), left ventricular systolic pressure (LVSP, (C), systemic vascular resistance index (SVRI, (D) and cardiac index (CI, (E) are shown. Values are mean ± SEM. *N *= 5–8. *P < 0.05 and **P < 0.01, versus baseline. (F and G) Representative hemodynamic measurement tracings showing effects of L‐NAME (30 mg/kg, iv) (F) and L‐canavanine (100 mg/kg, iv) injections (G) on systemic arterial pressure (SAP) and RVP in Sugen5416/hypoxia/normoxia exposed rats at the 8‐week time point.

To investigate the functional vascular role of basal EDNO activity, acute hemodynamic effects of intravenous L‐NAME were examined in normal and SU/Hx/Nx rats (1, 3, 5, and 8‐weeks after Sugen5416 injection). Five minutes after the baseline measurements, L‐NAME (30 mg/kg, total volume of 0.2–0.3 mL) was injected intravenously, and hemodynamic parameters were continuously monitored. We took hemodynamic parameter data in response to L‐NAME approximately 3 min after its injection when RVSP reached its plateau in normal and SU/Hx/Nx rats at the 1‐, 3‐, and 5‐week time points. Because RVSP declined steeply after it reached its peak around 1 min after L‐NAME injection in all SU/Hx/Nx rats at the 8‐week time point (see Fig. 1E), hemodynamic data in response to L‐NAME in this group were collected when RVSP reached its peak. In order to assess iNOS‐dependent NO release, acute hemodynamic effects of L‐canavanine (100 mg/kg, total volume of 0.1–0.2 mL) were also tested in SU/Hx/Nx rats at the 8‐week time point.

#### Protocol 2. Assessment of stimulated NO release in the hypertensive pulmonary circulation

ACh (5.0 *μ*g/kg/min for 5 min div.) was injected intravenously in rats to assess hemodynamic effects of endothelium‐dependent vasodilation in SU/Hx/Nx rats at the 1‐ and 8‐week time points. We have tested acute effects of acetylcholine (3–15 *μ*g/kg/min, div.) in the 8‐week SU/Hx/Nx rats according to the protocols previously reported (Feddersen et al. 1986). In our preliminary study, we observed that 10–15 *μ*g/kg/min caused severe bradycardia, which prevented measurement of stable hemodynamic parameters. Therefore, we used 5 *μ*g/kg/min of acetylcholine in this study. To verify if the vasodilatory mechanisms of NO was intact in SU/Hx/Nx rats at the 1‐ and 8‐week time points, we also tested effects of SNP (an endothelium‐independent vasodilator/NO donor; 5.0 *μ*g/kg/min for 5 min div.). Hemodynamic parameters were measured before (baseline) and after ACh or SNP infusion. The doses of all pharmacological agents used in this study were based on the literature (Shirai et al. 2003; Schwenke et al. 2011).

### Immunohistochemical analysis

The left lobe of the lungs was inflated with phosphate‐buffered saline (PBS) containing 1% formalin plus 0.5% agarose via trachea at a constant pressure of 20 cm H_2_O and were then fixed in 10% formalin neutral buffer solution overnight. The left lobe was embedded in paraffin. The 5‐*μ*m thick slices obtained at the level of the hilum were subjected to immunohistochemical staining. All sections were incubated with primary antibody reactive to eNOS (1:300; #9572; Cell Signaling Technology, Danvers, MA) at 4°C overnight. Sections were then incubated with biotinylated secondary antibody prior to horseradish peroxidase‐labeled streptavidin. The pulmonary arteries, which appeared to be in a perpendicular cross section, were counted, and the intensity of immunostaining was graded semi‐quantitatively, as previously described (Giaid and Saleh 1995). Staining intensity was graded semiquantitatively from 0 to 3, with 0 representing the absence of any staining and 3 representing the maximal intensity. We carefully identified and counted pulmonary arteries based on their relationship with accompanying airways and examined 3 ranges of vessel size: small; less than 50 *μ*m, medium; 50 to 100 *μ*m, and large; more than 100 *μ*m (Toba et al. 2014). For each animal, at least 55 vessels were analyzed in a blinded manner.

### Immunoblot analysis of the expression of eNOS and Phospho‐eNOS

The lungs were frozen at −80°C and subsequently homogenized in 50 mmol/L 4‐(2‐hydroxyethyl)‐1‐piperazineethanesulfonic acid (HEPES), pH 7.4, 150 mmol/L NaCl, 0.5% (v/v) Nonidet P‐40, 1 mmol/L EDTA, 1 mmol/L dithiothreitol, 0.5 mmol/L Na3VO4, 10 mg/mL leupeptin, 10 mg/mL aprotinin, 5 mmol/L microcystin‐LR, 10 mmol/L calpain inhibitor, and 10 mmol/L 4‐aminidophenylmethane sulfonyl fluoride (Abe et al. 2010). The protein concentration of the lysate was determined with a Coomassie protein assay kit (Pierce, Rockford, IL) with bovine serum albumin as a standard. Equal amounts of total proteins (10 *μ*g) were separated on 12.5% (w/v) polyacrylamide gels for sodium dodecyl sulfate polyacrylamide gel electrophoresis and transferred to a polyvinylidene difluoride membrane (0.2 *μ*m pore size; Bio‐Rad, Hercules, CA). The membranes were blocked with 5% (w/v) skim milk (for eNOS) or 5% (w/v) bovine serum albumin (for phospho‐eNOS) in 20 mmol/L TrisHCl, pH 7.5, 150 mmol/L NaCl, and 0.05% (v/v) Tween 20 (Tween 20 containing Tris‐buffered saline) overnight at 4°C. The membranes were then incubated for 1 h at room temperature with primary antibodies: eNOS (1: 5000), phospho‐eNOS (1: 2000), which were diluted in immunoreaction enhancer solution (Can Get Signal; Toyobo, Osaka, Japan), followed by a 1‐h incubation with secondary antibodies conjugated to horseradish peroxidase (1: 2000) (Matsuo et al. 2015). The immune complexes were detected using an ECL select detection kit (GE Healthcare, Buckinghamshire, UK). Light emission was detected and analyzed with VersaDoc 5000 and the computer program Quantity One (Bio‐Rad). We measured the following protein levels: eNOS, phospho‐eNOS, and *β*‐actin. The following antibodies purchased from Cell Signaling Technology (Danvers, MA) were used: anti‐eNOS (#9572), anti‐phospho‐eNOS (#9571), and anti‐*β*‐actin (#E2710). This anti‐phospho‐eNOS antibody was reported to detect endogenous levels of eNOS only when phosphorylated at Ser1177 (Matsuo et al. 2015). The optical density of each band was normalized to that of the corresponding *β*‐actin band.

### cGMP measurement by enzyme immunoassay

The protein levels of cyclic guanosine monophosphate (cGMP) in lung tissues from the early (1‐week) and late (8‐week) time points were determined by the cGMP enzyme immunoassay (EIA) (Cayman Chemical, Ann Arbor, MI) (Lang et al. 2012). A 50‐mg sample of frozen lung tissue was homogenized in 0.5 mL 5% trichloroacetic acid (TCA). The supernatant was subjected to TCA extraction using 2.5 mL water‐saturated ether three times and the residual ether from the aqueous layer was removed by heating the sample to 70°C. Next, 50 *μ*L of five‐times diluted samples and standard solutions were incubated with 50 *μ*L of tracer and 50 *μ*L of antibody at 4°C overnight. After washing five times, plates were incubated with Ellman's solution for 2 h at room temperature with gentle shaking. The plates were read at a wavelength of 412 nm, and the standard curve was produced using the Cayman EIA Triple workbook (Cayman Chemical, Ann Arbor, MI). The sample cGMP concentration was determined (as pmol/mg tissue) using the equation obtained from the standard curve.

### Plasma cell‐free hemoglobin measurement

Plasma cell‐free hemoglobin (CFH) has been reported as a potent NO scavenger in patients with PAH (Brittain et al. 2014). We therefore analyzed the plasma CFH levels in SU/Hx/Nx rats at the 1‐week (early) and 8‐week (late) time points. Blood sample was immediately and carefully collected from carotid artery access with a 24‐gauge Angiocath catheter to avoid hemolysis, and then was centrifuged within 15 min at 1500 *g*, and the plasma fraction were immediately stored at −80°C. Plasma CFH was detected using spectrophotometric methods with the QuantiChrom Hemoglobin Assay Kit (BioAssay Systems) (Brittain et al. 2014).

### Biopterin measurement in lung tissues by ELISA

Previous reports have shown that increased dihydrobiopterin (BH_2_) levels cause eNOS uncoupling even in the absence of tetrahydrobiopterin (BH_4_) deficiency in rats (Noguchi et al. 2011), and that BH_4_/BH_2_ ratio may be even more important than the absolute BH_4_ levels as a marker of eNOS coupling (Takeda et al. 2009). We therefore assessed BH_4_ and its ratio to BH_2_ in lung tissue isolated from normal and the early (1‐week) and late (8‐week) SU/Hx/Nx rats using the Rat BH4 ELISA kit (Elabscience Biotechnology Co). Lung tissue was minced into small pieces and rinsed in ice‐cold PBS (0.01 mol/L, pH = 7.4) to remove excess blood thoroughly. A 40‐mg sample of frozen lung tissue was homogenized in 200 *μ*L PBS with a homogenizer on ice. After freeze‐thaw cycles, the homogenates are then centrifuged to collect the supernatant. 50 *μ*L of diluted samples and standard solutions were incubated with 50 *μ*L of Biotynylated Detection Ab working solution for 45 min at 37°C. After washing three times, plates were incubated with 100 *μ*L of HRP conjugate working solution for 30 min at 37°C. After washing five times, 90 *μ*L of Substrate Solution was added and incubated for about 15 min at 37°C, protecting from light. After adding 50 *μ*L of Stop Solution to each well, the plates were read at a wavelength of 450 nm with the use of Multiskan Go Basic (Thermo Fisher Scientific). The concentrations of BH_4_ and BH_2_ (pg/mg tissue) were calculated using the equation obtained from the standard curve.

### Statistical analysis

Values are shown as mean ± SE. ANOVA with Bonferroni post hoc test was used for comparisons among the experimental groups (time point) or Student's *t* test for comparisons before (baseline) and after L‐NAME, ACh or SNP administration. Differences were considered significant at *P *< 0.05.

## Results

### RV hypertrophy and baseline hemodynamic measurement data (Table 1)

Consistent with our previous report (Toba et al. 2014), RV/LV+S ratios in SU/Hx/Nx rats were significantly elevated at the 1‐week time point and increased to a maximum at the 8‐week time point. Similarly, right ventricular systolic pressure (RVSP) significantly increased from the 1‐week time point and appeared to reach its maximum at the 8‐week time point. Cardiac index (CI) tended to decrease from the 5‐week time point and significantly decreased to approximately 50% of normal at the 8‐week time point. Accordingly, total pulmonary vascular resistance index (TPRI) significantly increased from the 3‐week time point and reached its maximum at the 8‐week time point. In contrast, there were no significant increases in left ventricle systolic pressure (LVSP) and heart rate (HR) over time. No significant changes of systemic vascular resistance index (SVRI) were observed at any time point except for the 8‐week time point when a significant reduction in CI and increase in SVRI were observed.

**Table 1:** Body weight, right ventricular hypertrophy, and baseline hemodynamic parameters in normal and various stages of PAH rats

	Normal	Sugen5416/hypoxia/normoxia rats			
		1‐week	3‐week	5‐week	8‐week
BW, g	159.5 ± 2.2	159.6 ± 5.4	237.3± 12.1*	298.4 ± 8.4*	349.8 ± 19.1*
RV/(LV+S)	0.27 ± 0.01	0.43 ± 0.02*	0.48 ± 0.01*	0.62 ± 0.02*	0.77 ± 0.02*
HR, bpm	365.8 ± 10.1	342.0 ± 14.0	338.6 ± 15.2	328.8 ± 8.92	317.8 ± 12.5
RVSP, mmHg	30.7± 0.6	57.8 ± 4.8*	91.7 ± 9.9*	95.8 ± 5.9*	128.5 ± 3.8*
CI, mL/min/kg	331.5 ± 21.1	262.1 ± 19.9	247.9 ± 44.0	207.3 ± 28.4	161.8 ± 23.2*
TPRI, mmHg/(mL/min/kg)	0.1 ± 0.01	0.23 ± 0.03	0.42 ± 0.06*	0.49 ± 0.07*	0.86 ± 0.1*
LVSP, mmHg	102.3 ± 4.3	110.3 ± 7.8	122.7± 8.9	105.7 ± 7.1	123.3 ± 9.4
SVRI, mmHg/(mL/min/kg)	0.32 ± 0.03	0.43 ± 0.05	0.57 ± 0.09	0.55 ± 0.08	0.84 ± 0.11*

Baseline data in normal and Sugen5416/hypoxia/normoxia‐exposed rats 1, 3, 5, and 8 weeks after Sugen5416 injection. Values are mean ± SEM, *N*=5–8 for each group, BW, body weight; RV, right ventricle; LV, left ventricle; S, septum; HR, heart rate; RVSP, right ventricular systolic pressure; CI, cardiac index; TPRI, total pulmonary vascular resistance index; LVSP, left ventricular systolic pressure; SVRI, systemic vascular resistance index. *P < 0.05 versus Normal.

### Effects of L‐NAME on hemodynamics in normal and SU/Hx/Nx rats (Protocol 1)

To investigate the functional vascular roles of basal endothelium‐derived NO (EDNO) release, acute hemodynamic effects of the non‐selective NOS inhibitor, L‐NAME, were examined in normal and SU/Hx/Nx rats. Blockade of NOS with L‐NAME (30 mg/kg, iv) did not change RVSP, TPRI or CI in normal and SU/Hx/Nx rats at the 1‐week time point, while it increased RVSP and TPRI markedly in SU/Hx/Nx rats at the 3‐, 5‐, and 8‐week time points (Figs. 1A and B). Consistent with previous reports (Rees et al. 1990), L‐NAME caused a marked elevation in LVSP in normal rats. This is also true for PAH rats at the 1‐ and 3‐week time points, but the L‐NAME‐induced LVSP elevations disappeared in the later (5‐ and 8‐week) time points associated with marked reductions in CI (Figs. 1C–E). Suprisingly, L‐NAME caused a rapid elevation in RVSP followed by a profound reduction in CI, resulting in a marked increase in TPRI, leading to death in all rats tested at the 8‐week time point (Fig. 1F). Acute inhibition of iNOS by L‐canavanine (100 mg/kg, iv) had no significant effects on RVSP, TPRI, CI, LVSP, and SVRI at the 8‐week time point (Fig. 1G).

### Immunohistochemical analysis of endothelial NO (eNOS) expression in normal and SU/Hx/Nx rats

**Figure 2:**
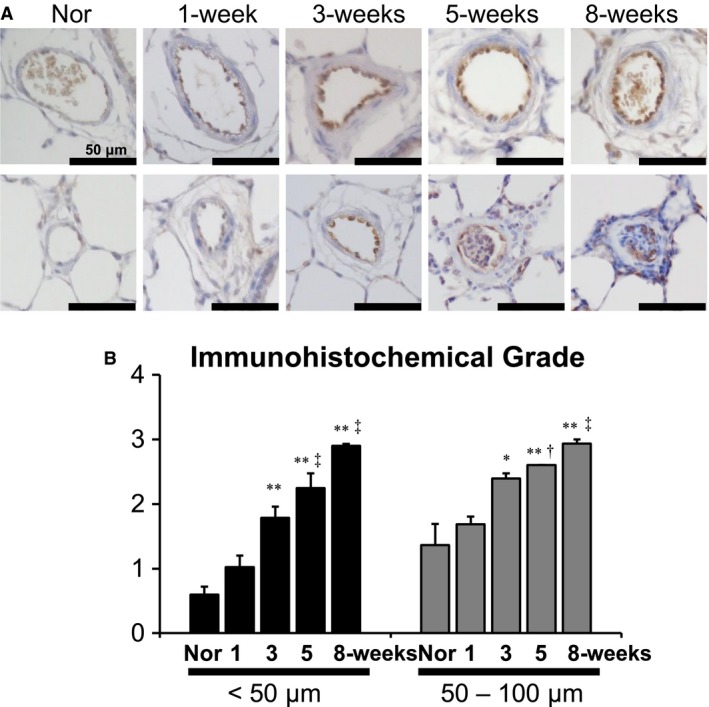
Immunoreactivity of endothelial nitric oxide synthase (eNOS) in pulmonary arteries of normal (Nor) and Sugen5416/hypoxia/normoxia‐exposed PAH rats. (A) Representative photomicrographs of eNOS‐stained pulmonary arteries at the 1‐, 3‐, 5‐, and 8‐week time points after the Sugen5416 injection (upper: 50–100 *μ*m of mediuim arteries, lower: <50 *μ*m of smaller arteries). Scale bars indicate 50 *μ*m. (B) Black, gray and white bars indicate immunoreactivity of eNOS in the innermost layer cells of smaller (<50 *μ*m) and medium (50–100 *μ*m) pulmonary arteries, respectively. The determination of immunohistochemical grade was described in method section. *N *= 4, each. *P < 0.05 and **P < 0.01, versus normal; ^†^ *P* < 0.05 and ^‡^ *P* < 0.01, versus 1‐week; ^§^ *P* < 0.01, versus 3‐week.

To investigate protein expression levels of eNOS in pulmonary arteries, we assessed a total of 1560 vessels in 20 left lobes from five groups of rats. Mean vessel numbers/lobe examined were, 62, 76, 72, 89, and 90, respectively, for normal, 1‐, 3‐, 5‐, and 8‐week time point PAH rats. In agreement with our previous report (Toba et al. 2014), we observed a time‐dependent increase in luminal occlusion by neointimal and plexiform lesions (data not shown). The parenchyma showed no apparent morphologic abnormalities. Immunohistochemical analyses revealed positive immunostaining for eNOS in the innermost layer cells of pulmonary arteries in all rats examined (Fig. 2A). As shown in Figure 2B, semiquantitative analyses of the immunohistochemical staining indicated that eNOS expression in smaller (<50 *μ*m) and medium (50–100 *μ*m) sizes of pulmonary arteries in SU/Hx/Nx rats was markedly increased from the 3‐week to 8‐week time points. No remarkable increases in eNOS expression were found in larger (>100 *μ*m) sizes of pulmonary arteries (data not shown).

### Immunoblot analysis of eNOS and its phosphorylation in normal and PAH rats

**Figure 3:**
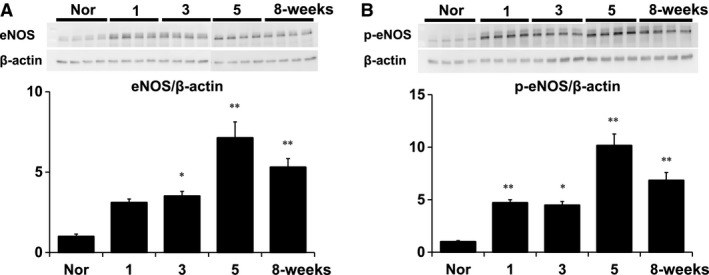
Protein levels of (A) eNOS and (B) phophorylated eNOS (p‐eNOS) in whole lungs isolated from normal (Nor) and the 1‐, 3‐, 5‐, and 8‐week time points Sugen5416/hypoxia/normoxia‐exposed PAH rats. Upper panels show actual immunoblots for eNOS, p‐eNOS and *β*‐actin. Lower panels show calculated densitometric ratios of eNOS/*β*‐actin and p‐eNOS/*β*‐actin. Values are means ± SEM of *N* = 4, each. *P < 0.05 and **P < 0.01 versus normal.

To examine activity of eNOS, we measured protein expression levels of eNOS and phosphorylated eNOS in lungs isolated from normal and SU/Hx/Nx rats at the 1‐, 3‐, 5‐, and 8‐week time points. In agreement with a previous report (Rafikova et al. 2013), protein levels of eNOS were increased at the 3‐, 5‐, and 8‐week time points compared with normal (Fig. 3A). The levels of phosphorylated eNOS, a marker of increased eNOS activity (Matsuo et al. 2015), were enhanced in the lungs at the 1–, 3‐, 5‐, and 8‐week time points (Fig. 3B).

### Effects of ACh and SNP on hemodynamics at the early and late stages of PAH (Protocol 2)

**Figure 4:**
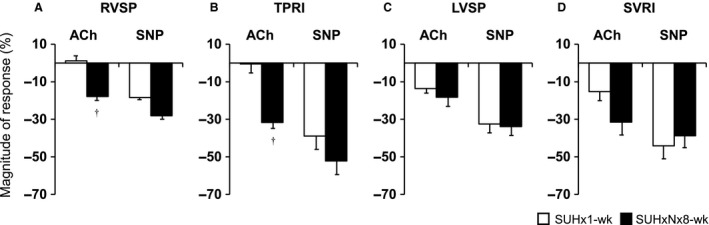
Effects of acetylcholine (ACh; 5 *μ*g/kg/min) and sodium nitroprusside (SNP; 5 *μ*g/kg/min) on hemodynamic parameters in the early (1‐week, white column) and the late (8‐week, black column) stages of Sugen5416/hypoxia/normoxia (SU/Hx/Nx)‐exposed PAH rats. (A) Right ventricular systolic pressure (RVSP). (B) Total pulmonary vascular resistance index (TPRI). (C) Left ventricular systolic pressure (LVSP). (D) Systemic vascular resistance index (SVRI). Percentage changes were expressed as a difference in values between baseline and after ACh or SNP divided by each baseline value. Values are mean ± SEM. *N* = 9–12. ^†^ *P* < 0.01 versus SUHx 1 week.

To assess hemodynamic effects of endothelium‐dependent and ‐independent pulmonary vasodilations, we measured acute hemodynamic effects of ACh and SNP in catheterized SU/Hx/Nx rats at the early (1‐week) and late (8‐week) time points. Compared with each baseline value, intravenous infusion of ACh (5 *μ*g/kg/min) significantly reduced RVSP (−24.9 ± 2.1 mmHg, Fig. 4A and Table 2) and TPRI (−0.34± 0.08 mL/min/kg, Fig. 4B and Table 2) at the 8‐week time point. ACh, however, induced no significant reductions in either RVSP or TPRI at the 1‐week time point (Figs. 4A, B, and Table 2). In stark contrast, intravenous infusions of the endothelium‐independent but NO‐mediated vasodilator, SNP (5 *μ*g/kg/min), reduced both RVSP and TPRI at the 1‐week as well as 8‐week time points (Fig. 4 and Table 2). Both agents similarly and significantly reduced in LVSP and SVRI at both time points (Figs. 4C, D, and Table 2).

**Table 2:** Effects of acetylcholine and sodium nitroprusside on hemodynamic parameters in early (1‐week) and late (8‐week) time points of Sugen5416/hypoxia/normoxia‐exposed PAH rats

		Baseline	ACh (5 *μ*g/kg/min)	SNP (5 *μ*g/kg/min)
1‐week	RVSP, mmHg	70.1 ± 3.8	70.7 ± 3.7	53.1 ± 3.5*†
	TPRI, mmHg/(mL/min/kg)	0.29 ± 0.03	0.29 ± 0.03	0.19 ± 0.02*
	LVSP, mmHg	119.7 ± 4.7	103.2 ± 3.7*	81.1 ± 1.1**, †
	SVRI, mmHg/(mL/min/kg)	0.50 ± 0.03	0.42 ± 0.03	0.29 ± 0.02**, †
8‐week	RVSP, mmHg	139.4 ± 5.6	114.4 ± 4.7*	103.7 ± 7.1**
	TPRI, mmHg/(mL/min/kg)	0.99 ± 0.08	0.65 ± 0.05**	0.60 ± 0.08**
	LVSP, mmHg	102.7 ± 4.9	82.9 ± 4.3*	73.2 ± 6.3**
	SVRI, mmHg/(mL/min/kg)	0.73 ± 0.06	0.48 ± 0.05**	0.38 ± 0.04**

Effects of acetylcholine (ACh; 5 *μ*g/kg/min) and sodium nitroprusside (SNP; 5 *μ*g/kg/min) on hemodynamics. Values are mean ± SEM. *N*=9–12 for each group. RVSP, right ventricular systolic pressure; TPRI, total pulmonary vascular resistance index; LVSP, left ventricular systolic pressure; SVRI, systemic vascular resistance index;

*P < 0.05 and **P < 0.01 versus Baseline, ^†^ *P* < 0.05 versus ACh.

### Effects of L‐NAME on lung tissue cGMP levels at the early and late stages of PAH

**Figure 5:**
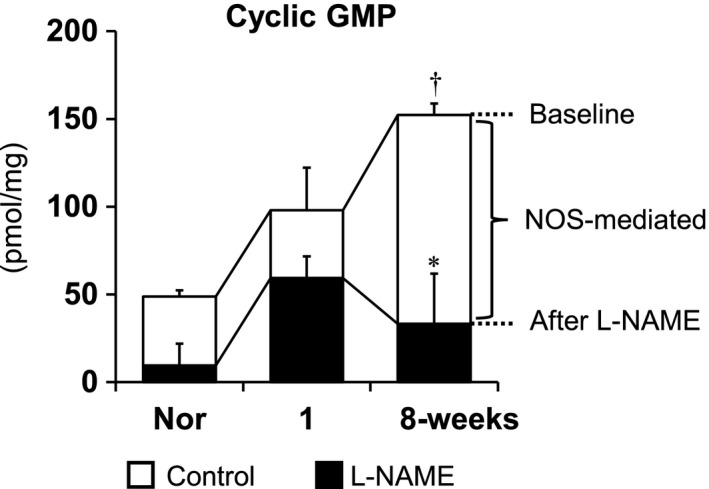
Effects of *N*_*ω*_‐nitro‐ L‐arginine methyl ester hydrochloride (L‐NAME, 30 mg/kg, iv) on cGMP concentration in lung tissue isolated from normal (Nor), the early (1‐week), and the late (8‐week) stages of Sugen5416/hypoxia/normoxia‐exposed PAH rats. Values which subtracted that of after L‐NAME (black columns) from baseline indicate NOS‐mediated cGMP production (white columns). Values are mean ± SEM. *N* = 4, each. † *P* < 0.01, versus normal. *P < 0.01, versus each baseline.

To determine NOS‐mediated cGMP production, we measured cGMP levels in the whole lung tissues from normal and SU/Hx/Nx rats at the 1‐week (early) and 8‐week (late) time points with and without addition of the NOS inhibitor L‐NAME (30 mg/kg, iv). The baseline (without L‐NAME) level of cGMP was significantly higher in lungs from SU/Hx/Nx rats at the 8‐week time point than that of normal (Fig. 5). Addition of L‐NAME significantly reduced the cGMP level only in lungs from SU/Hx/Nx rats at the 8‐week time point, but not in lungs from normal or SU/Hx/Nx rats at the 1‐week time point (Fig. 5). These results suggested that there was a significant amount of NOS‐mediated cGMP production (determined by its levels of baseline minus those of after addition of L‐NAME) only in lungs from SU/Hx/Nx rats at the 8‐week time point.

### Plasma cell‐free hemoglobin (CFH) measurements at the early and late stages of PAH

**Figure 6:**
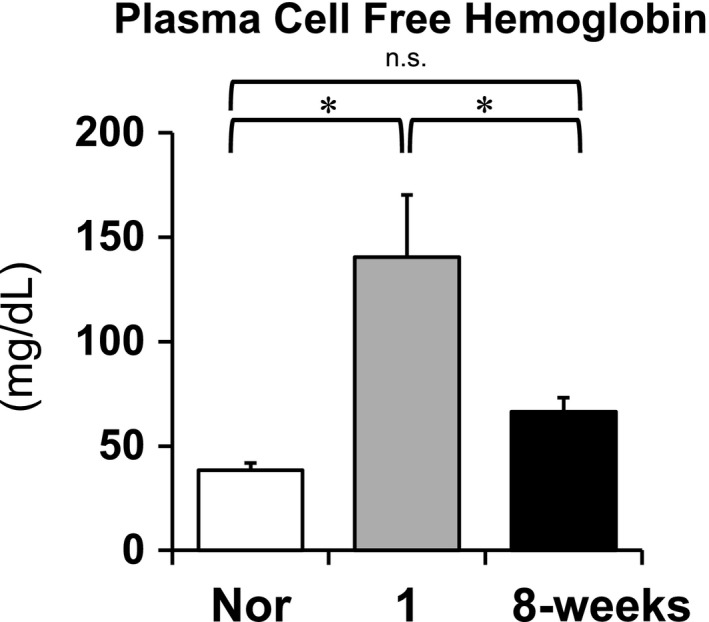
Plasma levels of cell‐free hemoglobin from normal (Nor), the early (1‐week), and the late (8‐week) stages of Sugen5416/hypoxia/normoxia‐exposed PAH rats. Values are mean ± SEM. *N* = 10–12. *P < 0.01 versus 1‐week. n.s. indicates not statistically significant.

To assess NO scavenging activity in PAH, baseline plasma CFH levels (a NO scavenger) in normal and SU/Hx/Nx rats at the 1‐week (early) and 8‐week (late) time points were measured. As shown in Figure 6, plasma CFH levels were significantly elevated in the early PAH rats compared with normal. The elevated CFH decreased to normal levels in the late‐stage PAH rats.

### BH_4_ and BH_2_ measurements in lung tissues at the early and late stages of PAH

**Figure 7:**
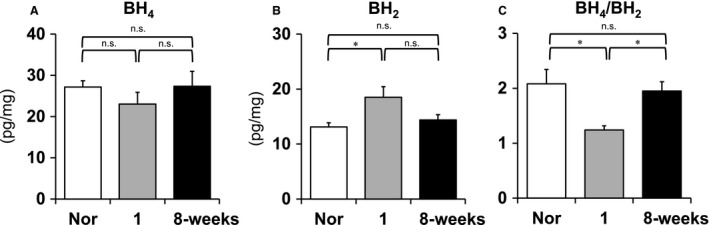
Levels of tetrahydrobiopterin (BH_4_) and dihydrobiopterin (BH_2_) in lung tissue isolated from normal (Nor), the early (1‐week), and the late (8‐week) stages of Sugen5416/hypoxia/normoxia‐exposed PAH rats. BH_4_ levels (A), BH_2_ levels (B), and BH_4_/BH_2_ ratio (C) are shown. Values are means ± SEM. *N* = 7, each. *P < 0.05 versus 1‐week. n.s. indicates not statistically significant.

We also examined a marker of eNOS coupling, biopterin levels, in the whole lung tissues of normal and Su/Hx/Nx rats at the 1‐week (early) and 8‐week (late) time points. Dihydrobiopterin (BH_2_) levels significantly increased in lungs from SU/Hx/Nx rats at the 1‐week time point compared with normal controls (Fig. 7A), whereas there were no significant differences in tetrahydrobiopterin (BH_4_) levels among normal and the early and late SU/Hx/Nx rats (Fig. 7B). Consequently, the BH_4_/BH_2_ ratio decreased at the 1‐week time point compared with other time points (Fig. 7C).

## Discussion

The major findings of this study are that blockade of NOS with L‐NAME (but not with the iNOS inhibitor L‐canavanine) caused marked increases in the already elevated RVSP at 3, 5, and 8 weeks after the initiation of the SU/Hx/Nx‐induced PAH in rats that simulate the human PAH phenotype histologically as well as hemodynamically (Abe et al. 2010; Toba et al. 2014). The increases in RVSP in response to L‐NAME were apparently caused by active pulmonary vasoconstriction since they were not accompanied by increases in cardiac output. In contrast, NOS inhibition did not increase the elevated RVSP nor decrease the CI at an earlier time point (1 week after the initiation) of this PAH model. It has previously been shown that Rho kinase‐mediated vasoconstriction contributes markedly to the high RVSP and TPRI throughout the PAH process, that is, from the early (1‐week) to the late (13‐week time point) in SU/Hx/Nx rats (Oka et al. 2007; Toba et al. 2014). It is therefore speculated that at late time points removal of EDNO by L‐NAME likely enhanced/unmasked the Rho kinase‐mediated vasoconstriction to further elevate RVSP. On the other hand, although there is Rho kinase–mediated vasoconstriction, NO inhibition did not increase RVSP at the early time point (Fig. 1), indicating that EDNO fails to attenuate the pulmonary vasoconstriction. We also observed that ACh and SNP induced similar and significant reductions in RVSP and TPRI in the late time point (8‐week) SU/Hx/Nx rats, whereas SNP but not ACh decreased RVSP at the early time point (1‐week) animals (Fig. 4 and Table 2). Taken together, these results suggest that, however, pulmonary EDNO is functionally inactive at the early time point, EDNO activity is restored and suppresses vigorous pulmonary vasoconstriction in severe established PAH. The results also indicate a greater contribution of a vasoconstriction component in the pathophysiology of occlusive PAH than previously recognized.

We measured lung tissue expression of phosphorylated eNOS (p‐eNOS) as eNOS activity and serum cGMP levels as a surrogate marker of NO production (Figs. 3 and 5) (Griffith et al. 1988; Preston et al. 2004; Galiè et al. 2005; Matsuo et al. 2015). As shown in Figure 3, the time‐dependent and similar increases in total and phosphorylated eNOS expressions suggest that most of the eNOS expression is in the active form in the established PAH. Because cGMP can also be produced by other than NOS activation, we determined the NOS‐mediated cGMP production as its baseline level minus that after addition of L‐NAME. We found in SU/Hx/Nx rats at the 1‐week time point that while eNOS activity was increased (Fig. 3), there was no increase in NOS‐mediated cGMP production (Fig. 5). Considering our observation that EDNO was not functional (i.e., NOS inhibition did not further increase the elevated RVSP) and ACh did not induce any pulmonary vasodilation, our findings suggest that NO bioavailability is impaired in lungs from SU/Hx/Nx rats at this early time point. We further found that CFH levels increased and BH_4_/BH_2_ ratio decreased in these lungs. These findings indicate that increased NO scavenging and eNOS uncoupling may be involved in the impaired NO bioavailability. In contrast, at the 8‐week time point, both eNOS activity and NOS‐mediated cGMP production were increased, indicating that the NO‐cGMP pathway activity is indeed upregulated at the late time point. The normalization of CFH levels and BH_4_/BH_2_ ratio may be at least partly responsible for the recovery of the impaired NO bioavailability at the 8‐week time point. Thus, the hemodynamic effects of L‐NAME on RVSP matches the NOS‐mediated cyclic GMP data in the early and late time points.

The finding that the NO‐cGMP pathway is upregulated in the rats with established PAH disagrees to some extent with the prevailing concept that endothelial dysfunction, particularly impaired NO bioavailability, is a pivotal factor in the progression of PAH (Tuder et al. 2009; Mam et al. 2010). However, a careful literature search surprisingly finds that the evidence supporting this concept is not solid (Klinger 2007). For instance, in addition to several opposing reports against the concept (Mason et al. 1998; Cremona et al. 1999), most supportive data have been derived from samples from end‐stage PAH patients at the time of autopsy or lung transplantation (Mason et al. 1998; Do e et al. 2009; Tuder et al. 2009), which may not reflect the status at earlier stages of the disorder. The only limited data available for assessment of NO availability during early stages of PAH are those of exhaled NO. There are supportive reports that NO levels in exhaled gas from PAH patients are lower than those of healthy controls (Archer et al. 1998; Kaneko et al. 1998; Girgis et al. 2005), but the interpretation of these reports has been questioned because there is the possibility that sources of NO other than the pulmonary vasculature could significantly be involved in the NO levels in exhaled gas (Cremona et al. 1999). There is also a recent report that measurements of exhaled NO levels do not differ between scleroderma associated PAH and healthy subjects (Cao et al. 2016).

An interesting and striking finding of this study is that in Su/Hx/Nx rats at the 8‐week time point, RVSP values reached very high levels (>150 mmHg) immediately after addition of L‐NAME followed by profound falls in CI, resulting in death of all SU/Hx/Nx rats due most likely to acute RV pump failure. These results suggest that at the late time point when severe PAH is established hemodynamically (high RVSP with decreased cardiac index) and histologically (fully developed neointimal occlusive lesions in small pulmonary arteries) (Toba et al. 2014), EDNO is still active and protecting against further deterioration of pulmonary hypertension, that is, increase in pulmonary vascular resistance and RV afterload. The observation of NOS inhibition‐induced acute deterioration of pulmonary hypertension, may explain mechanisms of some critical conditions observed in PAH patients. For example, it has been reported that pulmonary endothelial damage may be involved in marked elevations of PVR frequently observed in congenital heart disease associated PAH patients at the time of open‐heart surgery with cardiopulmonary bypass (Wessel et al. 1993; Winterhalter et al. 2010). If our current findings are true in PAH patients, and EDNO is actively opposing vigorous vasoconstriction in these patients, then endothelial damage and the accompanying decrease in EDNO bioavailability during the surgery would result in severe deterioration of pulmonary hypertension. Another example is the rebound phenomenon observed in PAH patients when continuous NO inhalation therapy is abruptly discontinued (Atz et al. 1996). It has been reported that inhibition of NOS by NO plays a major role in the mechanism of this rebound phenomenon (Oka et al. 1996; Sheehy et al. 1998). Again, if EDNO is active and suppressing pulmonary vasoconstriction, then inhibition of NOS by exogenous NO would lead to acute deterioration of pulmonary hypertension when NO inhalation is discontinued.

There are limitations in this study including the indirect measurement of endothelial NO bioavailability due to technical difficulty of real time measurements of NO levels in vivo. Also, possible gender differences (Rafikova et al. 2013) were not investigated in this study and this issue should be addressed in future studies.

In conclusion, this study demonstrated in a rat model of PAH that closely mimics the human PAH phenotype that although NO bioavailability was impaired initially, it was restored and even enhanced over time as PAH progressed and the restored EDNO activity moderated severe pulmonary vasoconstriction to keep RVSP and RV afterload at lower levels. These results suggest that impaired NO bioavailability can be restored during PAH progression, and that the contribution of a vasoconstrictive component in the pathophysiology of PAH may be greater than recognized. Because severe PAH deterioration occurs when EDNO activity is inhibited in established stages of PAH, it would be of importance in future studies to identify a way to maintain, or even enhance, EDNO activity to avoid not only acute but also, perhaps more importantly, chronic deterioration of PAH.

## Conflict of Interest

K.Abe. worked in a department endowed by Actelion Pharmaceuticals Japan., and received a research grant from Mochida Pharmaceutical Co., Ltd. K. Saku. received a research grant from Actelion Pharmaceuticals Japan. K.Sunagawa. works in a department endowed by Actelion Pharmaceuticals Japan., received a research funding from Actelion Pharmaceuticals Japan. H. Tsutsui. received honoraria from Daiichi Sankyo, Inc., Otsuka Pharmaceutical Co., Ltd., Takeda Pharmaceutical Company Limited, Mitsubishi Tanabe Pharma Corporation, Boehringer Ingelheim Japan, Inc., Novartis Pharma K.K., Bayer Yakuhin, Ltd., Bristol‐Myers Squibb KK, and Astellas Pharma Inc., and research funding from Actelion Pharmaceuticals Japan, Daiichi Sankyo, Inc., and Astellas Pharma Inc.
